# Ectopic expression of the *PISTILLATA* homologous *MdPI* inhibits fruit tissue growth and changes fruit shape in apple

**DOI:** 10.1002/pld3.51

**Published:** 2018-04-14

**Authors:** Jia‐Long Yao, Juan Xu, Sumathi Tomes, Wei Cui, Zhiwei Luo, Cecilia Deng, Hilary S. Ireland, Robert J. Schaffer, Andrew P. Gleave

**Affiliations:** ^1^ The New Zealand Institute for Plant & Food Research Limited Auckland New Zealand; ^2^ Key Laboratory of Horticultural Plant Biology (Ministry of Education) Huazhong Agricultural University Wuhan China; ^3^ School of Biological Sciences The University of Auckland Auckland New Zealand

**Keywords:** floral organs, fruit development, MADS‐box, *Malus* x *domestica*, *PISTILLATA*, transgenic plants

## Abstract

Fruit shape represents a key trait that consumers use to identify and select preferred cultivars, and although the manipulation of this trait is an opportunity to create novel, differentiated products, the molecular mechanisms regulating fruit shape are poorly understood in tree fruits. In this study, we have shown that ectopic expression of *Malus domestica PISTILLATA (MdPI),* the apple ortholog of the floral organ identity gene *PISTILLATA (PI),* regulates apple fruit tissue growth and shape. *MdPI* is a single‐copy gene, and its expression is high during flower development but barely detectable soon after pollination. Transgenic apple plants with ectopic expression of *MdPI* produced flowers with white sepals and a conversion of sepals to petals. Interestingly, these plants produced distinctly flattened fruit as a consequence of reduced cell growth at the basipetal position of the fruit. These altered sepal and fruit phenotypes have not been observed in studies using *Arabidopsis*. This study using apple has advanced our understanding of PI functions outside the control of petal and stamen identity and provided molecular genetic information useful for manipulating fruit tissue growth and fruit shape.

**Significance statement**

PISTILLATA (PI) and its homologs are mostly known to control the identity of petals and stamens in different plant species. This study has shown that ectopic expression of MdPI regulates apple fruit growth and shape, thus has advanced our understanding of PI and provided molecular genetic information useful for manipulating fruit shape.

## 1 INTRODUCTION

In flowering plants, various floral tissues contribute to fruit formation across species resulting in different fruit types (Spjut, 1994). In species with a superior ovary, such as grape and tomato, the berry fruit develops from the ovary tissue alone (Gillaspy, Bendavid, & Gruissem, 1993). In species with an inferior ovary, such as apple and pear, the pome fruit develops from the ovary and hypanthium tissues. The hypanthium surrounding the apple ovary is hypothesized to consist of the fused bases of the sepals, petals and stamens, and contributes tissue to the fruit flesh cortex while the ovary develops into the fruit core (Pratt, 1988).

As fruit is derived from floral organs, the genes regulating floral organ growth may also play important roles in controlling fruit development. Genes regulating floral organ development have been classified into four different classes by function, according to the proposed ABCE model (Krizek & Fletcher, 2005; Weigel & Meyerowitz, 1994). In summary, for *Arabidopsis*, the class A genes *APETALA1* (*AP1*) and *AP2* specify sepal formation; the class A genes and two class B genes [*APETALA3* (*AP3*) and *PISTILLATA* (*PI*)] together specify petal formation; *AP3* and *PI* together with the class C gene *AGAMOUS* (*AG*) are required for stamen formation; and *AG* alone controls carpel formation (Weigel & Meyerowitz, 1994). The class E *SEPALLATA* genes are required for the proper development of all four whorls of floral organs. A mutant plant for all four *SEPALLATA* genes (*sep1, sep2, sep3, sep4*) produces flowers consisting of reiterating whorls of leaf‐like organs (Krizek & Fletcher, 2005). All these class A, B, C, and E genes, except for *AP2*, belong to the highly conserved MADS‐box gene family. The role of these floral organ genes in regulating fruit development depends on their fruit structures or the floral tissue types contributing to fruit growth (Yao, Tomes, Xu, & Gleave, 2016; Yao et al., 2015).

*AP3* and *PI* are single‐copy genes in *Arabidopsis*. They are specifically expressed in petals and stamens during their development (Goto & Meyerowitz, 1994; Jack, Brockman, & Meyerowitz, 1992). Loss‐of‐function mutations of either *PI* or *AP3* produce flowers that contain two whorls of sepals and double numbers of carpels, but without stamens or petals (Goto & Meyerowitz, 1994; Jack et al., 1992). Ectopic expression of *PI* or *AP3* alone in *Arabidopsis* does not significantly change flower development, while ectopic expression of *PI* and *AP3* together converts sepals to petals, and carpels to stamens (Krizek & Meyerowitz, 1996). The functions of PI and AP3 are dependent on the coexistence of the two proteins forming a bifunctional heterodimer that activates genes involved in the control of numerous developmental processes required for organogenesis and represses key regulators of carpel formation (Wuest et al., 2012). In many species, there is a single copy of the *PI* homolog, but several copies of *AP3* as a result of gene duplication events (Lee & Irish, 2011). The single‐copy *PI* homolog has maintained its petal‐ and stamen‐specific expression pattern, in apple (Tanaka, Wada, Komori, Bessho, & Suzuki, 2007; Yao, Dong, & Morris, 2001) and grape (Sreekantan, Torregrosa, Fernandez, & Thomas, 2006), but some of the duplicated *AP3* paralogs have developed new expression specificities, such as sepal and fruit tissue expression in apple (Kitahara, Ohtsubo, Soejima, & Matsumoto, 2004; van der Linden, Vosman, & Smulders, 2002) and grape (Poupin et al., 2007). Ectopic expression of *VvPI* in grape inhibits fruit flesh tissue growth resulting in fleshless berries (Fernandez, Chaib, Martinez‐Zapater, Thomas, & Torregrosa, 2013). This is likely due to the expression of *AP3* homologs in the berry and therefore the formation of a PI/AP3 heterodimer.

Apple floral organ MADS‐box genes have been cloned and identified based on high sequence homology to the *Arabidopsis* ABCE classes of genes. These genes include two A class genes, *MdMADS5* (*MdAP1*) (Kotoda et al., 2002; Yao, Dong, Kvarnheden, & Morris, 1999) and *MdMADS2* (Sung, Yu, & An, 1999), three B class genes, *MdPI* (Yao et al., 2001), *MdMADS13* (van der Linden et al., 2002), and *MdTM6* (Kitahara et al., 2004), three C class genes, *MdMADS10* (Yao, Dong et al., 1999), *MdMADS14* and *15* (van der Linden et al., 2002), and five E class genes, *MdMADS4* (Sung, Yu, Nam, Jeong, & An, 2000), *MdMADS6*,* MdMADS7*,* MdMADS8,* and *MdMADS9* (Yao, Dong et al., 1999). After full genome sequencing, three more E class genes have been identified, *MdMADS18*,* MdMADS104,* and *MdMADS118* (Ireland et al., 2013). The apple floral organ genes have similar expression patterns to those of *Arabidopsis* homologs (Kitahara et al., 2004; Kotoda et al., 2002; van der Linden et al., 2002; Mimida et al., 2011; Sung et al., 1999, 2000; Tanaka et al., 2007; Yao, Dong et al., 1999), and overexpression of *MdPI* can rescue *Arabidopsis pi* mutants (Tanaka et al., 2007). They have similar functions in regulating floral organ development as the *Arabidopsis* genes, but play additional roles in regulating fruit development that are not known for *Arabidopsis* genes. In *Arabidopsis*, the *SHATTERPROOF* (*SHP*) 1 and 2 genes, most closely related to the class C gene *AG*, are shown to be important to silique development (Liljegren et al., 2000). But in apple, class B and E genes are also shown to be important to fruit development as the pome fruit is derived from both ovary and hypanthium tissues. For example, knockout of *MdPI,* in addition to producing pistillate flowers, confers parthenocarpic fruit development in apple (Yao et al., 2001), and antisense suppression *MdMADS8,* not only partially converts petals to sepals but also inhibits fruit flesh development and ripening (Ireland et al., 2013).

Although floral organ genes have been shown to influence fruit size, texture, and ripening, their function in altering fruit shape has not as yet been reported. In tomato, genes regulating fruit shape have been identified (Liu, Van Eck, Cong, & Tanksley, 2002; Rodriguez et al., 2011; Xiao, Jiang, Schaffner, Stockinger, & van der Knaap, 2008), but they do not belong to the ABCE classes of genes. In this study, we show that transgenic apple plants produce pistillate flowers and parthenocarpic fruit when *MdPI* expression is suppressed, and produce flowers with petal‐like sepals and fruit with a distinct new shape when *MdPI* is overexpressed.

## 2 EXPERIMENTAL PROCEDURES

### 2.1 RNA‐Seq and data analysis

Three biological replicates from whole flowers at balloon (5 days before full bloom) and full bloom stages, and one mixed sample each of leaf, root, and fruit at 5, 36, and 132 DAP (days after pollination) were collected from six trees of *Malus x domestica* “Royal Gala” and preserved in liquid nitrogen. RNA was isolated by rapid cetyltrimethyl ammonium bromide (CTAB) extraction (Chang, Puryear, & Cairney, 1993). Five μg total RNA was used to construct strand‐specific poly‐A RNA libraries as described by Ref. (Zhong et al., 2011). Libraries were sequenced by Macrogen (Republic of Korea) using HiSeq2000 sequencing system (Illumina) to produce reads per library ranging from 20‐27 million. Reads were aligned to apple gene models using Bowtie2, and reads per kilobase per million mapped reads (RPKM) were calculated in the statistical software R.

### 2.2 Transformation vector construction

For the production of apple transgenic plants, the pART7/pART27 (Gleave, 1992) binary vector system was used. The *MdPI* cDNA fragment between the P1 and P2 primers in pBluescript SK‐ (Yao et al., 2001) was cut out with *Kpn*1/*Bam*HI double digestion and cloned into the *Kpn*1/*Bam*H1 sites in pART7 between the CaMV35S promoter and *ocs* terminator in sense orientation. The *Not*I fragment containing the promoter–cDNA–terminator was then cloned into the *Not*I site of pART27 to form the pART27‐35S‐MdPI‐ocs construct (Figure S3b). This binary vector was then transferred into the *Agrobacterium tumefaciens* strain LBA4404 by electroporation for use in apple transformation.

### 2.3 Apple transformation

To produce transgenic apple plants, in vitro shoot cultures of *Malus* x *domestica* “Bolero” were established from bud wood collected from an orchard at The New Zealand Institute for Plant & Food Research Limited (PFR), Havelock North, New Zealand, using a previously described method (Yao, Cohen, Atkinson, Richardson, & Morris, 1995). Young leaves of the shoot cultures were used as explants in apple transformation experiments using the methods previously described (Yao, Tomes, & Gleave, 2013). The transgenic plants were grown on their own roots alongside wild‐type (WT) plants in a containment glasshouse using the methods previously described (Yao, Cohen, Van den Brink, & Morris, 1999). Flowers were pollinated with *Malus* x *domestica* “Granny Smith” pollen or covered with paper bags if pollination was not required.

### 2.4 Confirmation of transgenic apple plant

DNA was extracted from young leaves collected from WT and transgenic glasshouse‐grown plants using the DNeasy^®^ Plant Mini Kit (Qiagen). The DNA samples were used in PCR analyses to amplify a transgene fragment with primers 35SF and ocsR (Table S2). PCR products were analyzed on 0.7% agarose gels, and the fragments of expected size were purified using PureLink™ Quick Gel Extraction Kit (Invitrogen) before dispatch to Macrogen (Republic of Korea) for sequencing.

### 2.5 Quantitative RT‐PCR

For qRT‐PCR analysis of “Bolero” apple transgenic plants, total RNA was isolated from unopened balloons and 2‐week‐old fruit. To distinguish the expression of B class MADS‐box genes between floral and hypanthium tissues, RNA was isolated from dissected tissues of “Royal Gala” apple at four stages of floral development: Balloon, open flower, 2, and 8 days after pollination, as previously described (Ireland et al., 2013). RNA was isolated using the method developed for pine tree RNA extraction (Chang et al., 1993), analyzed using an Agilent 2100 bioanalyzer (Agilent Co, Ltd, USA) to determine RNA concentration and integrity, and then treated with DNaseI. For each RNA sample, 1 μg RNA was used for cDNA synthesis using the Quantitect^®^ Reverse Transcription Kit (Qiagen) according to the manufacturer's instructions. Using the cDNA as templates, qRT‐PCR was carried out using LightCycler^®^ 480 (Roche Diagnostics) following previously described procedures (Drummond et al., 2009). PCR primers for the reference control genes Actin and EF‐1α, and test genes *MdPI*,* MdTM6*,* MdMADS13* are listed in Table S2. Significant differences between gene expression levels were determined using analysis of variance (ANOVA) in SAS (version 9.2), with a *p* value <.05.

### 2.6 Light microscopy analysis

Samples were fixed in FAA (4% formalin/50% alcohol/5% acetic acid in water to 100%). Samples were washed twice in 50% ethanol, then dehydrated through a graded ethanol series at 2‐hr intervals, then 50:50 ethanol/xylene (2 hr), two changes of xylene (3 hr each), 1:1 wax/xylene (3 hr), and two changes of paraffin wax (12 hr each), and embedded in Paraplast wax (Oxford Labware, www.kendellhq.com) Sections of 10 μm thickness were cut using a Leitz 1512 microtome (Leica.www.leica-microsystems.com), placed on positively charged glass slides and dried overnight in a slide dryer. Sections were dewaxed in two changes of xylene (5 min each), followed by two changes of absolute ethanol and then air‐dried. The sections were stained with Safranin–Fast Green. Sections were photographed using an Olympus Vanox AHT3 microscope (Olympus Optical, www.olympus-global.com). Fruit cell area was measured using the IMAGEJ software (https://imagej.nih.gov/ij/), and 30 cells were measured at each of the three positions of a fruit.

## 3 RESULTS

### 3.1 Apple has a single *PISTILLATA* ortholog specifically expressed in flowers

An extensive search of the apple genome sequence using BLAST with other plant homologs found a single copy of the *PI* gene, which has been previously reported as *MdPI* (Yao et al., 2001) (Figure S1), with gene model number MDP0000286643 and located at 24 Mb on chromosome 8 (Velasco et al., 2010). Due to the relatively recent genomewide duplication in the apple genome, two paralog copies are usually identified for most floral genes (Ireland et al., 2013; Tian et al., 2015). A chromosome fragment loss after the genomewide duplication may explain the single‐copy status of *MdPI*. On the other hand, four *AP3* homologs were found in the apple genome (Figure S1), two with identical coding sequences but located on different chromosomes, consistent with a recent gene duplication.

Transcriptomic analysis of “Royal Gala” apple tissues by RNA‐seq technology revealed that *MdPI* transcript levels were very high in whole flowers at balloon (600 RPKM) and open flower (300 RPKM) stages, but were greatly reduced in fruit soon after pollination with an RPKM value of 3 at 5 DAP (days after pollination), and were not detected in fruit from 36 DAP through to maturity (Figure S2, Table S1). Relative transcript levels of the *AP3* homologs were variable between the different genes and between tissues. In general, RNA levels of the *AP3* homologs were lower than *MdPI* in flowers (Table S1). Transcripts for class A, C, and E genes were detected in both flowers and fruit tissues (Figure S2, Table S1). For example, high levels of transcripts were detected in both flower and fruit tissues for the class A gene *MdMADS5*, class C genes *MdMADS14* and *MdMADS15*, and class E genes *MdMADS6, MdMADS1/8*, and *MdMADS3/7*. These are consistent with previous results of northern and qRT‐PCR analyses (Ireland et al., 2013; van der Linden et al., 2002; Yao, Dong et al., 1999).

**Figure 1:**
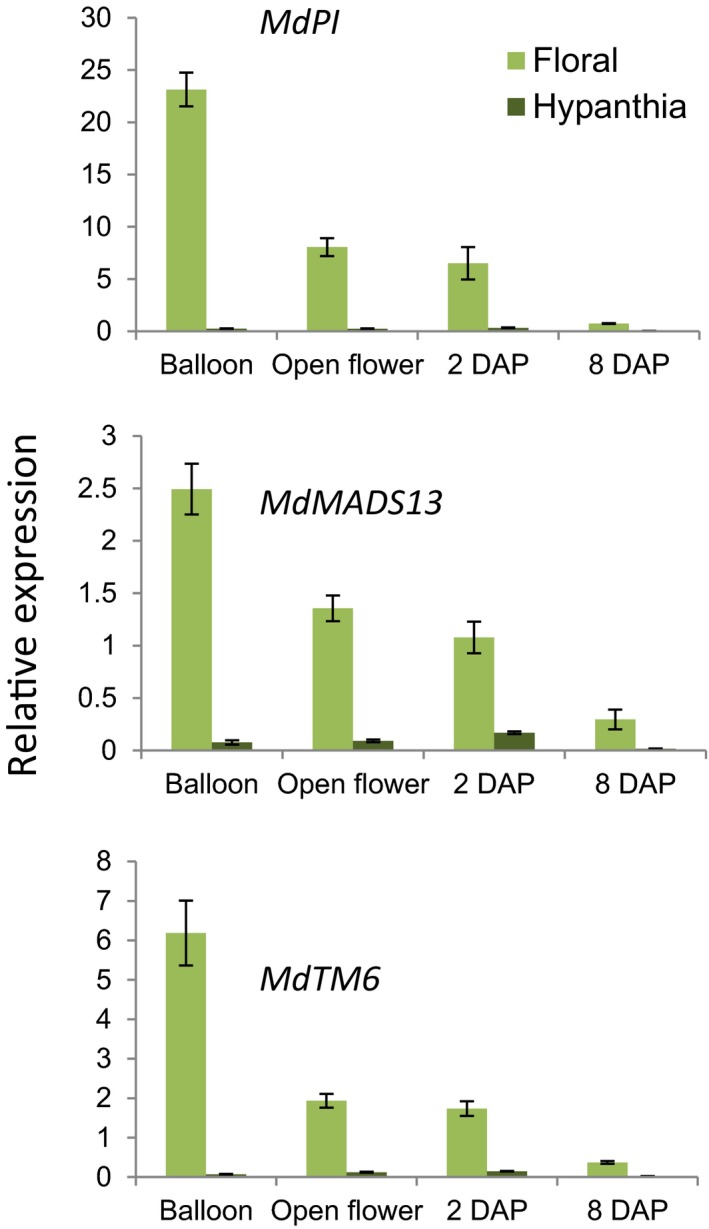
Quantitative PCR analyses of expression of *MdPI*,* MdMADS13,* and *MdTM6* in floral and hypanthium tissues of wild‐type (WT) “Royal Gala” apple plants. RNA from floral and hypanthium tissues were amplified using three primer pairs for *MdPI*,* MdMADS13,* and *MdTM6* respectively. Error bars represent the standard deviation of three independent PCR mixtures

We further analyzed B function gene expression using qRT‐PCR on two types of tissues dissected from “Royal Gala” flowers: the hypanthium (base of the flower cut in half and ovary tissue removed) and combined floral tissues (sepals, petals, stamens, and ovary tissues that were separated from the hypanthium). We found that expression levels of *MdPI, MdAMDS13,* and *MdTM6* were high in floral tissues at balloon stage and were reduced at open flower stage and at 2 DAP (Figure 1). The very low level of expression of *MdMADS13* and *MdTM6* at 8 DAP was likely from sepal and ovary tissues as petal and stamen tissues were no longer present at 8 DAP. The expression levels of *MdPI* and *MdTM6* were hardly detectable in the hypanthium from the balloon stage to 8 DAP. *MdMADS13* expression was detected at very low level in the hypanthium across all the stages we analyzed. Given the hypothesis that hypanthium consists of the fused bases of the sepals, petals, and stamens, it is interesting to note that the B function genes were weakly expressed in apple hypanthium. In this study, although “Royal Gala” was used in analyses of floral MADS‐box gene expression, “Bolero” apple was used in transgenic experiments because it is a dwarf cultivar. As there are no differences in floral or fruit structures between “Royal Gala” and “Bolero”, it is not unreasonable to assume that any differences in the expression of floral organ and fruit developmental genes between the two cultivars would be minimal. Nevertheless, our interpretations using data derived from different cultivars should be treated with some degree of caution.

### 3.2 Production and molecular analyses of apple transgenic plants

Six “Bolero” apple transgenic plants (*mdpi‐1* to *mdpi‐6*) were produced using a *35S‐MdPI* gene construct to overexpress the *MdPI* gene and grown to produce flowers and fruits in a glasshouse. The phenotypes of these transgenic plants are summarized in Table 1. The *mdpi‐1* and *mdpi*‐*2* plants produced flowers with white sepals, short anther filaments, and flattened fruit, whereas the *mdpi‐3* plant was largely normal except for short anther filaments. The *mdpi‐4*,* mdpi*‐*5* and *mdpi*‐*6* plants produced pistillate flowers and parthenocarpic fruits.

**Table 1:** Summary of transgenic phenotypes (apple cultivar “Bolero”)

Transgenic line	Flower	Fruit	*MdPI* expression
*mdpi‐1*	White sepals, short stamen filaments	Flattened	Upregulated
*mdpi‐2*	White sepals, short stamen filaments	Flattened	Upregulated
*mdpi‐3*	Short stamen filaments	Normal	No significant change
*mdpi‐4*	Pistillate	Parthenocarpic	Downregulated
*mdpi‐5*	Pistillate	Parthenocarpic	Downregulated
*mdpi‐6*	Pistillate	Parthenocarpic	Downregulated

DNA fragments of the *MdPI* transgene were amplified from the six transgenic lines *mdpi‐1* to *mdpi‐6* using primers that anneal to the 35S promoter and *ocs* terminator. The amplified fragments contained the full‐length transgene of *MdPI* as the primers used flanked the *MdPI* cDNA. No fragment was amplified from the WT apple or water control (Figure S3a). The amplified *MdPI* transgene fragments from three transgenic lines (*mdpi‐1*,* mdpi*‐*2,* and *mdpi*‐*4*) representing two of the three phenotypes observed were found to be identical in sequence. This indicated that the phenotypic differences were not due to any sequence variation in the *MdPI* transgene, but more likely due to differences in the transgenic *MdPI* expression levels.

**Figure 2:**
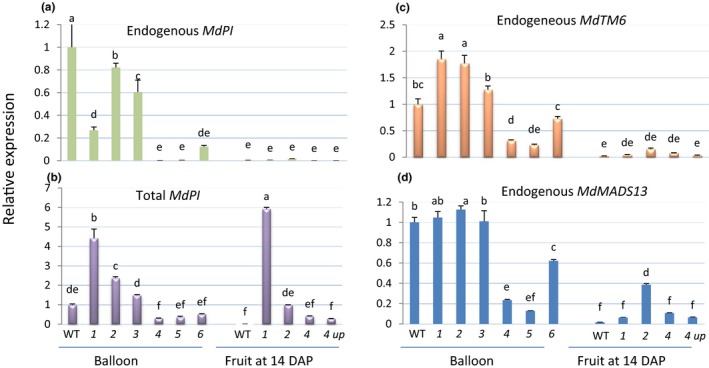
Quantitative PCR analyses of expression of *MdPI*,* MdMADS13*, and *MdTM6* in wild‐type (WT) and transgenic “Bolero” apple plants. RNA from unopened flowers and 2‐week‐old fruit of WT and transgenic lines (*mdpi‐1* to *6*) were amplified using four primer pairs for endogenous *MdPI* (a), total (endogenous plus transgene) *MdPI* (b), *MdTM6* (c), and *MdMADS13* (d), respectively. Significant differences between gene expression levels were analyzed using Duncan's test in SAS (version 9.2), and the *p* value was set as .05, three replications. *4up*: fruit developed from unpollinated flowers of the *mdpi‐4* transgenic line. DAP: days after pollination

To determine the relative expression levels of *MdPI* in the transgenic and WT plants, qRT‐PCR analyses were carried out using RNA extracted from unopened flowers and 2‐week‐old fruit (Figure 2). Using primers specifically designed to detect mRNA transcribed from the endogenous *MdPI* gene, *MdPI* transcripts were not detected in the flower samples of *mdpi*‐*4* and *mdpi‐5* (Figure 2a), indicating a complete suppression of gene expression, and transcript levels were reduced to varying degrees in the other four transgenic flower samples when compared to WT samples (Figure 2a). In 2‐week‐old fruit, no endogenous *MdPI* transcripts were detected in pollinated WT, *mdpi‐1*,* mdpi*‐*2,* or *mdpi*‐*4* fruit or unpollinated *mdpi‐4* fruit (Figure 2a) indicating that endogenous *MdPI* is not expressed in young fruit, a finding that is consistent with the RNA‐seq analysis (Figure S1) and a previous study (Yao et al., 2001). Using primers designed to detect transcripts from both the endogenous and transgenic *MdPI*, mRNA levels were found to be significantly higher in *mdpi‐1* and *mdpi‐2* plants than in WT in both the unopened flower and 2‐week‐old fruit samples (Figure 2b), indicating ectopic transgene expression. In contrast to *mdpi*‐*1* and *mdpi‐2*, the total mRNA levels of *MdPI* in *mdpi*‐*4*,* mdpi‐5,* and *mdpi‐6* were reduced compared to WT at the unopened flower stage. However, at the 2‐week‐old fruit stage, the *MdPI* mRNA levels of *mdpi‐4* were not significantly different from those of WT irrespective of whether the flowers had been pollinated or not. The variations in levels of transgene expression detected here are consistent with those in previous reports, showing different levels of transgene expression in different transgenic plants even if they were produced using the same gene construct and transformation methodology (Meyer, 1995). In some cases, when the transcription levels of transgene reached a high threshold level, post‐transcription gene silencing is triggered to degrade the transcripts of both transgene and its endogenous homologous gene, known as cosuppression (Que, Wang, English, & Jorgensen, 1997). The reductions in endogenous *MdPI* transcript level in *mdpi*‐1 to *mdpi*‐*6* (Figure 2a) were likely the result of partial or complete cosuppression. Collectively, the above information led us to suggest that the white‐sepal phenotype of *mdpi*‐*1* and *mdpi*‐*2* is caused by overexpression of *MdPI* and the *pistillata*‐like phenotype in *mdpi*‐*4*,* mdpi*‐*5,* and *mdpi*‐*6* is caused by downregulation (cosuppression) of *MdPI*.

As PI protein forms a heterodimer with AP3 to perform its function (Wuest et al., 2012), we wanted to determine whether changes in *MdPI* expression had any effect on the expression of *AP3* homologs. Using qRT‐PCR analyses, we found that the mRNA levels of two *AP3* homologs, *MdTM6* and *MdMADS13*, were significantly reduced in the flowers of transgenic apple plants where the *MdPI* gene was silenced (*mdpi‐4* and *5*). However, overexpression of *MdPI* in *mdpi‐1* and *mdpi*‐*2* plants was concomitant with elevated expression of *MdTM6* while there was no change in the expression level of *MdMADS13* (Figure 2c and d). In 2‐week‐old fruits, the mRNA level of *MdMADS13* is significantly higher in *mdpi‐2* than in WT (Figure 2d). It has been reported in *Arabidopsis* that a knockout of one partner has a negative effect on the gene expression of another partner (Goto & Meyerowitz, 1994). In this study, *AP3* homologs in apple flowers were downregulated when *MdPI* was cosuppressed.

### 3.3 Overexpression of *MdPI* confers sepal to petal conversion and changes fruit shape in apple

**Figure 3:**
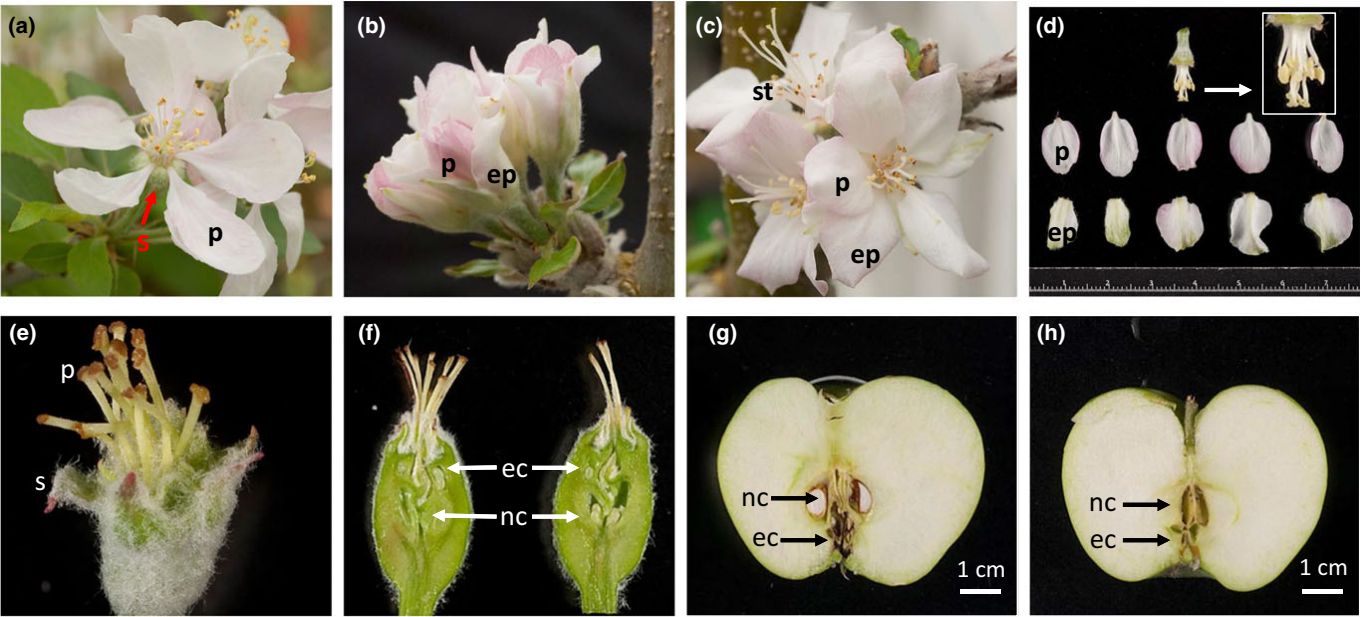
Flower and fruit of transgenic “Bolero” apple plants with overexpression or suppressed expression of *MdPI*. Photographs show opened flowers of wild‐type (WT) apple (a), unopened (b), opened (c), and dissected (d) flowers of transgenic line *mdpi‐1* overexpressing *MdPI*. Flower of transgenic line *mdpi*‐*4* shows no petals or stamens but increased numbers of sepals and styles (e), and two whorls of carpels (f). The flower may develop seeded fruit with pollination (g) or seedless fruit without pollination (h). S _= _sepal, p = petal, ep = ectopic petal, p = pistil, s = sepal, nc = normal carpels, ec = ectopic carpels

In WT “Bolero” flowers, the sepals were green and small with lots of trichomes (Figure 3a). In contrast, sepals in *mdpi*‐*1* (Figure 3b, c and d) and *mdpi‐2* transgenic plants were white to light pink in color, enlarged and without visible trichomes. This result suggests the conversion of sepals of *mdpi*‐*1* and ‐*2* plants to petals. This first whorl conversion was much stronger than the phenotype observed in *Arabidopsis* overexpressing *MdPI* (Tanaka et al., 2007) or *PI* (Krizek & Meyerowitz, 1996), where only the margin of sepals was converted to petals. In WT apple flowers, anther filaments were similar in length to styles (Figure 3a), while in *mdpi*‐*1*,* mdpi*‐*2,* and *mdpi*‐*3* plants, anther filaments were approximately half the length of styles (Figure 3c and d).

**Figure 4:**
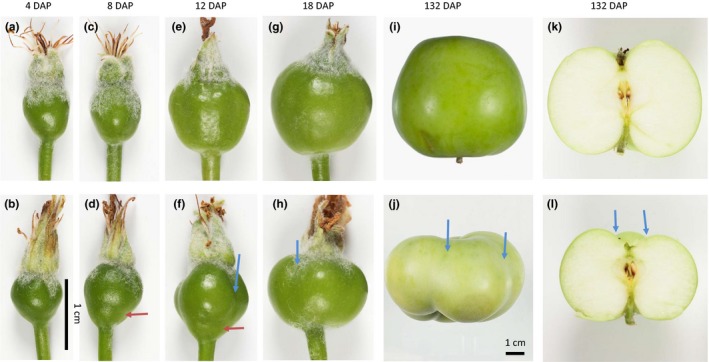
Fruit shape differences between wild‐type (WT) “Bolero” and *MdPI* overexpression apples at five developmental stages. The photographs show fruit of WT (a, c, e, g, i, k) and transgenic line *mdpi‐1* apple (b, d, f, h, j, l) at 4, 8, 12, 18, and 132 DAP (days after pollination). The longitudinal grooves (indicated using blue arrows) were retained on mature fruit. The transverse grooves (indicated by red arrows) were not on the mature fruit. The tissues below the transverse grooves did not continue to grow, which caused the flatten fruit phenotype at mature fruit stage. a to h are on the same scale, and i and j are on the same scale

Flowers of *mdpi*‐*1* and *mdpi‐2* developed fruit after hand pollination with “Granny Smith” apple pollen. These fruit showed an unusual shape (Figure 4, Figure S4), distinctively different from WT fruit and evident from the early stages of fruit development and through to fruit maturation. Starting from 8 DAP, the fruit surface showed grooves that were not observed on WT fruit. The grooves were distributed in two different orientations. Five longitudinal grooves were present along the side of the fruit from 12 DAP to fruit maturation (Figure 4f, h, j, l, and Figure S4b), corresponding to the five carpels. One transverse groove was present at the basipetal position of the fruit 8‐12 DAP (Figure 4d and f), but was no longer visible from 18 DAP to fruit maturation. From 18 DAP, the fruit of *mdpi*‐*1* and *mdpi‐2* plants was distinctively shorter than WT fruit with a very obvious flattened appearance at maturation (Figure 4). Fruit shape index, the ratio of maximum fruit height to maximum width (H/W), was significantly reduced in *mdpi*‐*1* and *mdpi‐2* fruit (Figure S4). The presence of grooves and the short and flattened fruit suggests that ectopic expression of *MdPI* suppressed localized fruit tissue growth.

**Figure 5:**
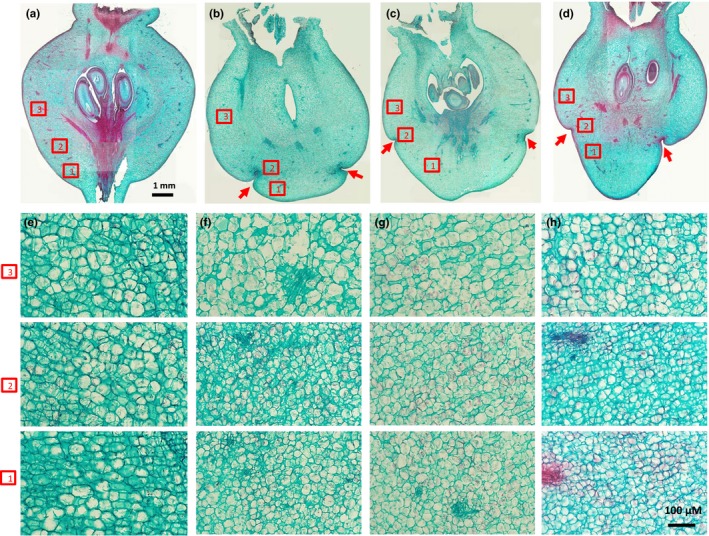
Fruit sections of wild‐type (WT) and *MdPI* overexpression (*mdpi*‐*1)* “Bolero” apples. Longitudinal sections of a WT fruit (a) and three different fruit of the *mdpi*‐*1* transgenic plant (b, c, d) at 12 DAP (days after pollination) stained with Safranin‐Fast Green. The transverse grooves at the basipetal position of *mdpi*‐*1* fruit are indicated by the red arrows. The areas marked with red squares in a, b, c, and d were photographed at higher magnification and present in e, f, g, and h, respectively. The numbers 1, 2, and 3 in the square represent the position in the fruit, as 1 = below the groove, 2 = at the groove, and 3 = above the groove. a to d are on the same scale, and e to h are on the same scale

**Figure 6:**
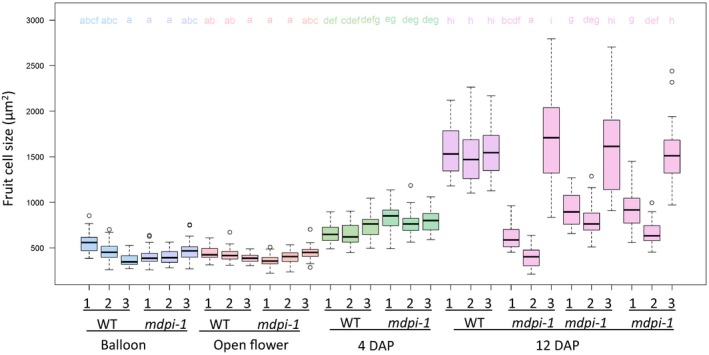
Hypanthium and fruit cell size of wild‐type (WT) and *MdPI* overexpression (*mdpi*‐*1)* “Bolero” apples. A box‐plot shows the cell size distribution in hypanthium of balloon and open flowers and in fruit at 4 and 12 DAP (days after pollination) of WT and *mdpi*‐*1* transgenic plants. The numbers 1, 2, and 3 in the *X*‐axis labels represent the positions in the fruit as the same as those described in Figure 5 and Figure S5, that is, 1 = below the groove, 2 = at the groove, and 3 = above the groove. Thirty cells were measured at each of the three positions of a fruit. Significant differences between means (*n* = 30) were analyzed using Tukey's HSD (honest significant difference) test with a single‐step multiple comparison procedure and the *p* value was set as .01

Stained longitudinal sections of *mdpi*‐*1* and WT fruit at 12 DAP showed clear groove traces at the basipetal position of the *mdpi*‐*1* fruit, but not of the WT fruit (Figure 5). These grooves were not present in WT fruit at any developmental stage (Figures 4 and 5, Figure S5). Three selected fruit of *mdpi*‐*1* at 12 DAP showed a different level of development of the basipetal groove (Figure 5b, c and d). The groove on the fruit in Figure 5b and d was deeper than that on the fruit in Figure 5c. The width of fruit tissue below the groove was sharply reduced for the fruit in Figure 5b and d, compared to the fruit tissue above the groove. By examining these sections at high magnification, we observed that fruit fleshy cells close to and below the groove were significantly smaller than those above the groove, (Figures 5 f‐h and 6), and the cells close to the groove were the smallest at 12 DAP (Figure 6). However, this difference in cell size was not observed among the corresponding three positions of *mdpi*‐*1* plant at three earlier stages, balloon, open flower stages, and 4 DAP, or among the corresponding three positions of WT plants at any of the four stages examined (Figures 5 and 6, Figure S5). Fruit cell size was significantly increased from 4 DAP to 8 DAP in WT fruit and in the acropetal position of *mdpi‐1* fruit, but did not increase at the basipetal position in *mdpi‐1* fruit (Figure 6). This indicated that the development of a flat *mdpi*‐*1* fruit started between 4 and 12 DAP by inhibiting fruit cell enlargement at the basipetal part of the fruit. Accurate counting of cell numbers in this region was not feasible as it was impossible to define comparable area or cell layers between the WT and the transgenic plant, or between the regions up and down the groove line. Collectively, these findings lead us to conclude that the flattened fruit occurs primarily because of inhibition of cell expansion at the basipetal position of the fruit, although we cannot exclude the possibility of reduced cell division and therefore reduced cell number.

### 3.4 Suppression of *MdPI* expression confers petal to sepal, and stamen to carpel conversion

WT apple flowers usually consist of five sepals, five petals, 9–20 stamens, and five carpels (Figure 3a). In contrast, flowers of *mdpi*‐*4* (Figure 3e and f), *mdpi‐5,* and *mdpi‐6* transgenic apples had no petals or stamens but two whorls of sepals and two whorls of carpels; thus, sepal number increased to 10 and the carpel number increased up to 15 (Figure 3). This result indicates floral organ conversion of petal to sepal, and stamen to carpel in these transgenic plants. Flowers of these transgenic lines were consistent with the phenotype of *pistillata* mutants of *Arabidopsis* (Goto & Meyerowitz, 1994) and apple *pistillata‐*like flowers of the apple cultivars “Rae Ime,” “Willington Bloomless,” and “Spencer Seedless.” These three apple cultivars are mutants with knockout of *MdPI* expression (Yao et al., 2001). Normal fruit development of WT “Bolero” flowers requires pollination and seed development. Flowers of *mdpi*‐*4*,* mdpi‐5,* and *mdpi‐6* developed seeded fruits after pollination with “Granny Smith” pollen (Figure 3g); when the flowers were covered with paper bags to prevent pollination, they developed seedless fruits (Figure 3h). The mature seedless fruit were similar in size to the seeded fruit (Figure 3g and h). Both seeded and seedless *mdpi*‐*4* fruits had a whorl of normal carpels and a whorl of ectopic carpels (Figure 3, Figure S6). These *mdpi*‐*4* fruits also had duplicated whorls of calyces that were the remnants of sepals, in contrast to the one calyx whorl of a normal WT apple. The feature of double whorls of calyces and carpels in the fruit of *MdPI* cosuppression transgenic apple plants was the same as that of the fruit of the apple mutant “Rae Ime” (Yao et al., 2001).

## 4 DISCUSSION

### 4.1 Class B MADS‐box genes regulate flower development in apple

We have demonstrated that suppression of *MdPI* expression in transgenic apple produced pistillate flowers with floral organ conversion of petals to sepals, and stamens to carpels. This floral organ conversion is identical to that observed in *Arabidopsis pi* mutants (Goto & Meyerowitz, 1994) and apple *mdpi* mutants (Yao et al., 2001). This strong *pi* mutant phenotype in apple is consistent with absence of gene function redundancy because there is only a single *MdPI* gene present in apple.

Ectopic expression of *MdPI* in transgenic apple fully converts sepals to petals. However, the ectopic expression of the *MdPI* or *PI* gene in *Arabidopsis* only converts the base and margins of sepals to petal tissue (Tanaka et al., 2007). Ectopic expression of both *PI* and *AP3* together converts sepals to petals and carpels to stamens (Krizek & Meyerowitz, 1996), indicating that the functions of PI and AP3 are dependent on the coexistence of the two proteins to form a heterodimer (Wuest et al., 2012). In *Arabidopsis*, there is a single copy of the *AP3* gene that is expressed at the base of sepals and throughout petals and stamens. The conversion of the base of sepals to petal cell types is a result of coexistence of the endogenous AP3 and transgenic PI (Krizek & Meyerowitz, 1996). The apple genome contains four copies of *AP3* homologs (Figure S1) that are expressed in all four types of floral organs, although the expression in sepals and carpels is weaker than in petals and stamens (Kitahara et al., 2004; van der Linden et al., 2002). The full conversion of sepals to petals is likely the result of coexistence of endogenous AP3 homologs and transgenic MdPI in the first whorl floral organs of apple. However, it is not clear why there is no carpel to stamen conversion although there is likely coexistence of endogenous AP3 homologs and transgenic MdPI in the fourth whorl floral organs of the transgenic apple.

### 4.2 Class B MADS‐box genes regulate fruit development in apple

Interestingly, ectopic *MdPI* expression in apple changed the fruit shape, to a distinctive flattened fruit, as a consequence of suppressed fruit tissue growth. Our analyses of the phenotype showed that localized fruit tissue growth was suppressed from early in fruit development, as indicated by the grooves formed in two different orientations on the fruit surface only a few days after fruit set. The suppression at the basipetal position of the fruit makes it short and flat, and the suppression on the sides of the fruit creates a pumpkin‐like shape with grooves on the side of the fruit. Histological analyses showed that at 12 DAP cells were smaller in the grooved region and in the fruit tissue below the groove toward the fruit stalk. The tissue size of this region was reduced in two of the three fruit analyzed. Suppression of cell expansion may be the key reason for reduced tissue growth between 4 and 12 DAP although we cannot rule out the possibility of inhibition of cell division. From 18 DAP to fruit maturation, the basipetal groove was no longer observed and the fruit became short and flat, suggesting complete inhibition of tissue growth in this region from 18 DAP by inhibiting both cell expansion and cell division.

Ectopic expression of *PI* in *Arabidopsis* does not change silique development (Krizek & Meyerowitz, 1996; Tanaka et al., 2007) probably because *AP3* is not expressed in the silique (Jack et al., 1992). In contrast, ectopic expression of *VvPI* in grape inhibits fruit flesh tissue growth resulting in fleshless, small berries (Fernandez et al., 2013), as one of *AP3* homologs, *VvTM6*, is expressed in the berry (Poupin et al., 2007) and therefore a PI/AP3 heterodimer is able to form. The overall transcript level of *AP3* homologs in apple fruit is relatively low, suggesting that these *AP3* transcripts may be specifically localized in the fruit thereby dictating the localization of the AP3 protein, subsequent formation of AP3/PI heterodimer, and suppression of fruit cell growth.

When *MdPI* expression was suppressed, apple fruit tissue growth in the absence of fertilization was promoted resulting in the production of parthenocarpic apple fruit, a phenotype that has been observed in apple *mdpi* mutants but not been observed in *Arabidopsis pi* mutants. There is evidence to suggest that AP3 and PI homologs suppress ovary and fruit development in grape, tomato, and orchid (*Phalaenopsis equestris),* and such suppression can be released by pollination (Dauelsberg et al., 2011; Mazzucato, Olimpieri, Siligato, Picarella, & Soressi, 2008; Tsai et al., 2005). Our results, together with those of studies on grape, tomato, and orchid, have advanced our understanding of the function of PI/AP3 outside petals and stamens, and beyond that revealed in studies using *Arabidopsis*.

The external characteristics of fruit appearance, such as shape, color, and size, represent key attributes that consumers use to identify and select preferred cultivars, and it has been suggested that the manipulation of these is a largely untapped opportunity to create novel, differentiated products (Gamble, Jaeger, & Harker, 2006). While color and size are often a focus in commercial breeding programs, there is good evidence that the manipulation of fruit shape also represents an opportunity to create visually distinct products that can be branded (Gamble et al., 2006). A practical example is provided by the way the shape of flat peaches is being used to differentiate them from conventional cultivars (Romeu, Sanchez, & Garcia‐Brunton, 2015). Genetic markers have been developed for the flat peaches and used in selection breeding seedlings (Picanol et al., 2013). Mapping‐based cloning approach has revealed that the candidate gene for the trait is possibly a LRR‐RLK protein kinase rather than a MADS‐box gene (Lopez‐Girona et al., 2017). QTL underlying apple fruit shape variations have been located on chromosome 11 using a mapping population derived from a “Jonathan”“Golden Delicious” cross (Cao et al., 2015; Chang et al., 2014). This is different from the location of *MdPI* that is on chromosome 8. Currently, most commercial apple cultivars are similar in shape, and the findings we have described here provide new molecular genetic information that could be used in genetic transformation of current apple cultivars to change their fruit shape. Also *Malus* germplasm resources could be exploited to identify accessions with different fruit shape index and determine whether there is a strong association between fruit shape index and DNA markers in the chromosomal region containing the *MdPI* gene. If such an association is determined, these markers can be used to accelerate the breeding of new fruit shape in apple to generate distinctive cultivars of higher commercial value.

## CONFLICT OF INTEREST

The authors declare no conflict of interests.
