# Supplementary figures and images for: Ectopic expression of the PISTILLATA homologous MdPI inhibits fruit tissue growth and changes fruit shape in apple
Source: Plant Direct. 2018 Apr 14;2(4):e00051. doi: 10.1002/pld3.51 (PMC6508508; doi:10.1002/pld3.51)

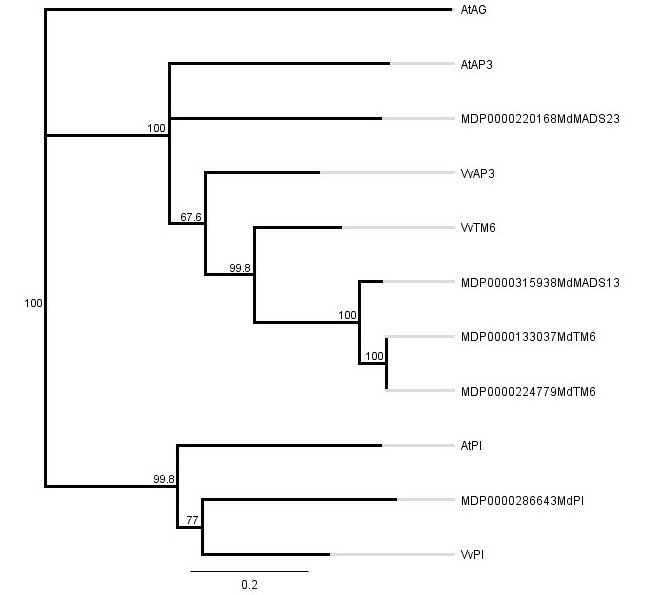

Supplement: Supplementary file 1 [file PLD3-2-e00051-s001.JPG]

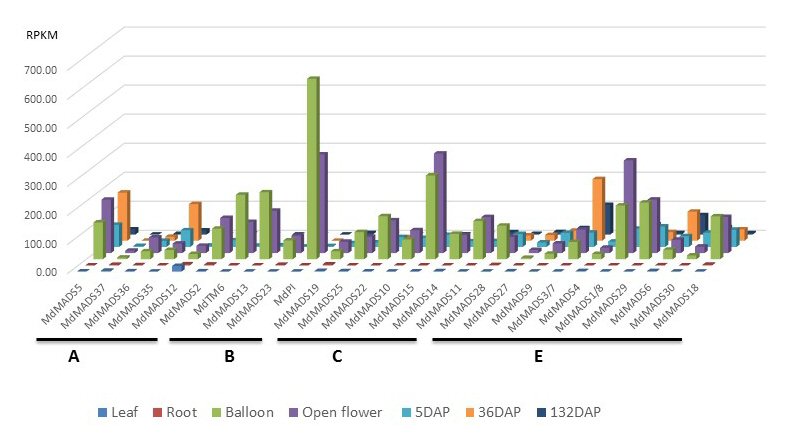

Supplement: Supplementary file 2 [file PLD3-2-e00051-s002.JPG]

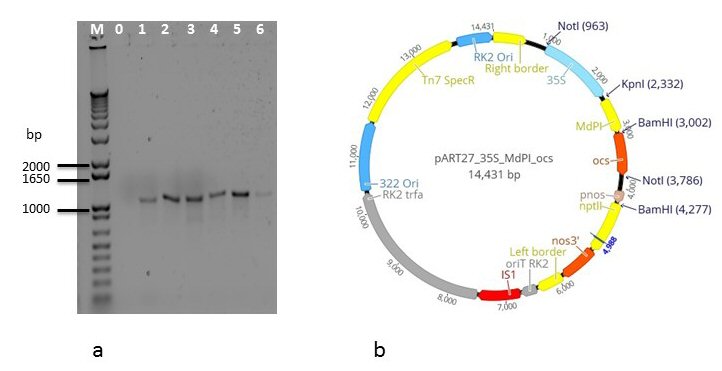

Supplement: Supplementary file 3 [file PLD3-2-e00051-s003.JPG]

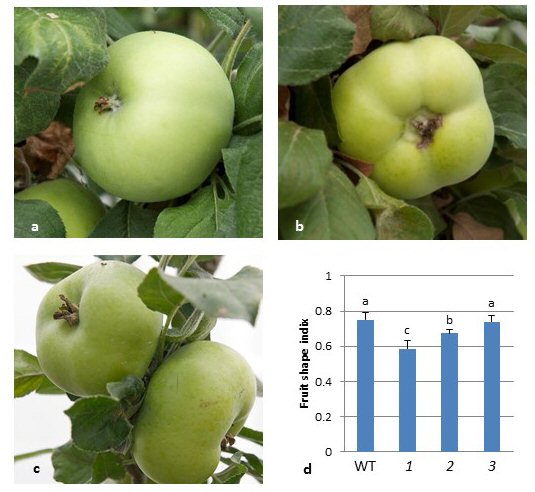

Supplement: Supplementary file 4 [file PLD3-2-e00051-s004.JPG]

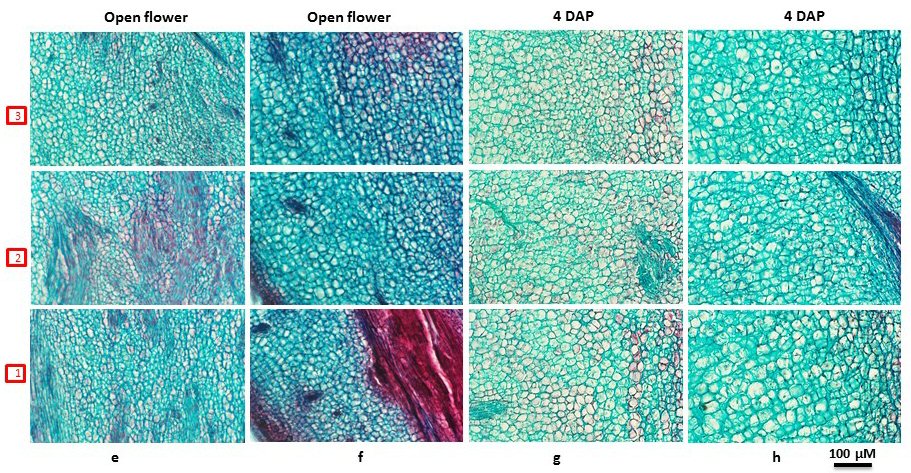

Supplement: Supplementary file 5 [file PLD3-2-e00051-s005.JPG]

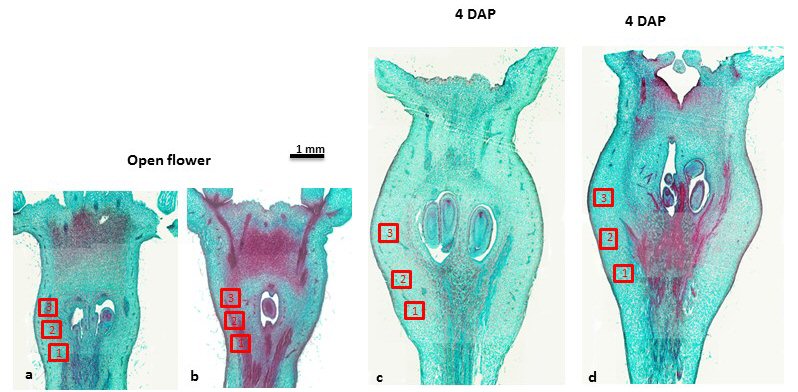

Supplement: Supplementary file 6 [file PLD3-2-e00051-s006.JPG]

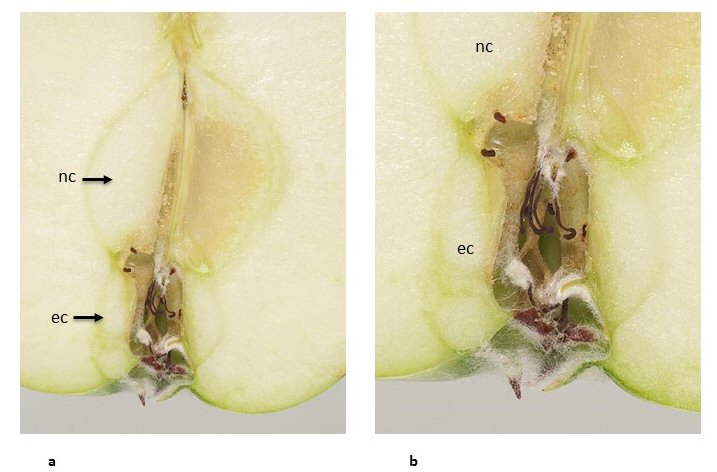

Supplement: Supplementary file 7 [file PLD3-2-e00051-s007.JPG]
