# Supplementary material for: Ectopic expression of the PISTILLATA homologous MdPI inhibits fruit tissue growth and changes fruit shape in apple
Source: Plant Direct. 2018 Apr 14;2(4):e00051. doi: 10.1002/pld3.51 (PMC6508508; doi:10.1002/pld3.51)
Supplement: Supplementary file 8 [file PLD3-2-e00051-s008.doc]

**Supplemental Tables**

**Table S1**. Apple floral MADS-box genes and their expression levels in seven different tissue types as determined by mRNA-seq analyses.

| **Gene class** | **Gene Name** | **Gene model ID** | **Gene expression level (RPKM)** | | | | | | |
| --- | --- | --- | --- | --- | --- | --- | --- | --- | --- |
|  |  |  | **Leaf** | **Root** | **Balloon** | **Open flower** | **5DAP** | **36DAP** | **132DAP** |
| a | *MdMADS5* | MDP0000013331 | 0.64 | 0.00 | 126.69 | 184.13 | 76.03 | 166.06 | 18.00 |
| a | *MdMADS37* | MDP0000132738 | 2.94 | 2.25 | 5.80 | 8.49 | 3.32 | 0.40 | 0.19 |
| a | *MdMADS36* | MDP0000218020 | 0.85 | 0.78 | 27.53 | 54.00 | 21.01 | 13.85 | 2.51 |
| a | *MdMADS35* | MDP0000269921 | 0.00 | 0.00 | 32.10 | 32.64 | 57.38 | 126.49 | 14.19 |
| a | *MdMADS12* | MDP0000278897 | 19.54 | 4.05 | 18.32 | 25.29 | 4.53 | 0.43 | 0.30 |
| a | *MdMADS2* | MDP0000289836 | 0.89 | 2.86 | 105.51 | 121.02 | 23.02 | 24.00 | 3.29 |
| b | *MdTM6* | MDP0000133037 | 0.00 | 0.00 | 222.27 | 107.49 | 4.22 | 0.06 | 0.00 |
| b | *MdMADS13* | MDP0000184294 | 0.00 | 0.00 | 230.75 | 145.92 | 5.67 | 0.06 | 0.00 |
| b | *MdMADS23* | MDP0000220168 | 0.23 | 1.96 | 64.64 | 59.69 | 1.39 | 0.50 | 0.00 |
| b | *MdPI* | MDP0000286643 | 0.00 | 0.00 | 621.58 | 339.87 | 3.04 | 0.00 | 0.00 |
| c | *MdMADS19* | MDP0000120269 | 2.28 | 3.50 | 27.52 | 39.32 | 13.08 | 4.98 | 5.18 |
| c | *MdMADS25* | MDP0000242009 | 1.37 | 0.00 | 93.78 | 56.27 | 15.58 | 16.13 | 0.96 |
| c | *MdMADS22* | MDP0000250080 | 0.66 | 0.00 | 148.33 | 112.85 | 33.83 | 12.50 | 0.75 |
| c | *MdMADS10* | MDP0000268317 | 0.00 | 0.00 | 67.97 | 78.75 | 31.15 | 10.87 | 0.69 |
| c | *MdMADS15* | MDP0000324166 | 0.63 | 0.00 | 288.70 | 342.88 | 41.53 | 18.27 | 0.79 |
| c | *MdMADS14* | MDP0000324259 | 1.26 | 0.00 | 87.67 | 62.29 | 19.55 | 19.08 | 1.35 |
| e | *MdMADS11* | MDP0000149676 | 0.00 | 0.00 | 131.73 | 123.90 | 20.04 | 15.50 | 8.19 |
| e | *MdMADS28* | MDP0000220008 | 0.00 | 1.37 | 115.80 | 55.39 | 44.71 | 18.39 | 2.23 |
| e | *MdMADS27* | MDP0000280009 | 0.78 | 0.00 | 4.92 | 10.79 | 15.29 | 19.71 | 8.91 |
| e | *MdMADS9* | MDP0000289858 | 0.00 | 0.18 | 19.13 | 33.31 | 48.31 | 34.89 | 21.95 |
| e | *MdMADS3/7* | MDP0000326390 | 0.00 | 0.16 | 59.61 | 81.01 | 49.37 | 212.51 | 102.02 |
| e | *MdMADS4* | MDP0000326906 | 1.99 | 0.00 | 18.30 | 18.44 | 18.51 | 26.09 | 9.95 |
| e | *MdMADS1/8* | MDP0000366022 | 0.00 | 0.15 | 185.11 | 319.27 | 62.69 | 45.08 | 34.94 |
| e | *MdMADS29* | MDP0000370413 | 0.00 | 0.00 | 195.35 | 184.16 | 70.45 | 29.98 | 5.16 |
| e | *MdMADS6* | MDP0000574222 | 1.90 | 0.42 | 33.18 | 46.07 | 36.99 | 100.30 | 66.92 |
| e | *MdMADS30* | MDP0000605482 | 0.00 | 0.00 | 13.00 | 22.56 | 49.07 | 35.61 | 25.21 |
| e | *MdMADS18* | MDP0000936232 | 0.00 | 2.54 | 147.88 | 124.38 | 59.41 | 39.17 | 5.01 |

**Table S2**. Primers used in this study.

| **Gene name** | **Primer name** | **Product size (bp)** | **Purpose** | **Sequence 5'-3'** |
| --- | --- | --- | --- | --- |
| 35S promoter | 35SF | 1093 | PCR | CAACCACGTCTTCAAAGCAA |
| ocs terminator | ocsR |  | PCR | TGTCGCTATAAACCTATTCAGCA |
| *MdTM6* | MdTM6F2 | 204 | RT-PCR | CTGCCAGTGAGAATCCACAA |
| *MdTM6* | MdTM6R2 |  | RT-PCR | AAGCAAGGCGGAGATCATGT |
| *MDMADS13* | MdMADS13F2 | 157 | RT-PCR | CCACAGTATGGTTATGAGGA |
| *MDMADS13* | MdMADS13R2 |  | RT-PCR | GGAGATCGTGCAGATGAGTA |
| *MdPI* | MdPIF2 | 167 | RT-PCR | TCGTCGACATGATGAGAGAC |
| *MdPI* | MdPIR1 |  | RT-PCR | CTGCTGCTGGTTGTTGTTGT |
| *MdPI* | MdPIF2 | 208 | RT-PCR | TCGTCGACATGATGAGAGAC |
| *MdPI* | MdPIR2 |  | RT-PCR | AGATTTGGCTGAATAGGCTG |
| *Actin* | EST324195RT-F | 150 | RT-PCR | GGGCTGTTTTCCCCAGTATT |
| *Actin* | EST324195RT-R |  | RT-PCR | TGCTAACAATCCCGTGTTCA |
| *EF-1α* | EST432952RT-F | 155 | RT-PCR | CGGCTTTACAAGGCACAAAT |
| *EF-1α* | EST432952RT-R |  | RT-PCR | AGTTCCACGACCCTGAATTG |

**Supporting figure legends**

**Figure S1**. Phylogenetic relationship of apple and *Arabidopsis* AP3-like and PI-like proteins.

Full-length protein sequences from apple (Genome Database for *Rosaceae*, [www.rosaceae.org](http://www.rosaceae.org/) ), grape (NCBI, <https://www.ncbi.nlm.nih.gov/>) and *Arabidopsis* (TAIR, [www.arabidopsis.org](http://www.arabidopsis.org/) ) were aligned with Geneious Alignment and used to compile the Neighbor-Joining tree using Geneious 10.0.3. *Arabidopsis* At4g18960.1 (AtAG) is included as an outgroup. Bootstrap values (shown on branches) were calculated using 500 datasets. Scale indicates 0.2 substitutions per site. Accession numbers are DQ979341 (*V. vinifera*, VvTM6). EF418603 (VvAP3), DQ988043 (VvPI), At3g54340.1 (AtAP3), At5g20240.1 (AtPI) and At4g18960.1 (AtAG).

**Figure S2.** mRNA-seq analysis of apple ABCE MADS-box genes in six tissue types of wild-type (WT) ‘Royal Gala’ apple.

Gene expression (RPKM) is shown for leaf, root and whole flowers at two development stages (floral balloon and open flower), and whole fruit at three development stages (5, 36 and 132 DAP; days after pollination).

**Figure S3.** PCR analysis of ‘Bolero’ transgenic plants and map of the gene construct used to produce these transgenic plants.

**a**, a DNA fragment approximately 1100 bp was amplified from the genomic DNA of six transgenic plants *mdpi-1* to *6* (1-6) but not from the DNA of the WT control plant (0) using primers binding to the 35S promoter and *ocs* terminator. M = 1 kb plus DNA ladder.

**b**, the map of the *MdPI* overexpression construct show that the *MdPI* cDNA sequence is cloned between the 35S promoter and ocs terminator in sense orientation in a binary vector containing the NPTII gene for kanamycin selection during plant transformation.

**Figure S4**. Flattened fruit of transgenic ‘Bolero’ apple plants with *MdPI* over-expression.

Fruit of wild-type (WT) (**a**), transgenic line *mdpi-1* (**b**) and *mdpi-2* (**c**) at 132 DAP (days after pollination). Mean fruit shape index of WT, *mdpi-1, 2 and 3* at 112 DAP (**d**), n = 11, 10, 4 and 18 for WT, *mdpi-1, 2 and 3* respectively. The error bars represent standard deviations. *mdpi-1* and *2* were over-expressing *MdPI*, but *mdpi-1* was not (Figure 4). Significant differences between fruit shape indexes were analysed using Duncan’s test in SAS (version 9.2), and the P value was set as 0.05, n = 10.

**Figure S5.** Flower and fruit sections of wild-type (WT) and *mdpi-1* ‘Bolero’ apple.

Longitudinal sections of flowers at open flower stages, and fruit at 4 DAP (days after pollination) of WT (**a**, **c**,) and transgenic *mdpi-1* (**b**, **d**) were stained with Safranin-Fast Green. The areas marked with red squares in **a** to **d** were photographed at higher magnification and present in **e** to **h** respectively. The number 1, 2, and 3 in the square correspond to the positions labelled in Figure 7 and 8, 1 = below the groove, 2 = at the groove, and 3 = above the groove. **a** to **d** are on the same scale, and **e** to **h** are on the same scale.

**Figure S6**. Fruit of ‘Bolero’ apple transgenic line *mdpi-4* contains a whorl of normal carpels (nc) and a whorl of ectopic carpels (ec).

**b** is the higher magnification image of **a**.
